# 3-Ethyl-4-[(*E*)-(4-fluoro­benzyl­idene)amino]-1*H*-1,2,4-triazole-5(4*H*)-thione

**DOI:** 10.1107/S1600536812015346

**Published:** 2012-04-18

**Authors:** S. Jeyaseelan, H. C. Devarajegowda, R. Sathishkumar, Agnes Sylvia D’souza, Alphonsus D’souza

**Affiliations:** a Department of Physics, Yuvaraja’s College (Constituent College), University of Mysore, Mysore 570 005, Karnataka, India; b Solid State and Structural Chemistry Unit, Indian Institute of Science, Bangalore, Karnataka, India; c Department of Chemistry, St. Philomena’s College, Mysore 570 015, Karnataka, India

## Abstract

In the title compound, C_11_H_11_FN_4_S, the dihedral angle between the 1,2,4-triazole ring and the benzene ring is 25.04 (12)° and an intra­moleuclar C—H⋯S inter­action leads to an *S*(6) ring. In the crystal, inversion dimers linked by pairs of N—H⋯S hydrogen bonds generate *R*_2_^2^(8) loops.

## Related literature

For a related structure and background references, see: Devarajegowda *et al.* (2010 ▶).
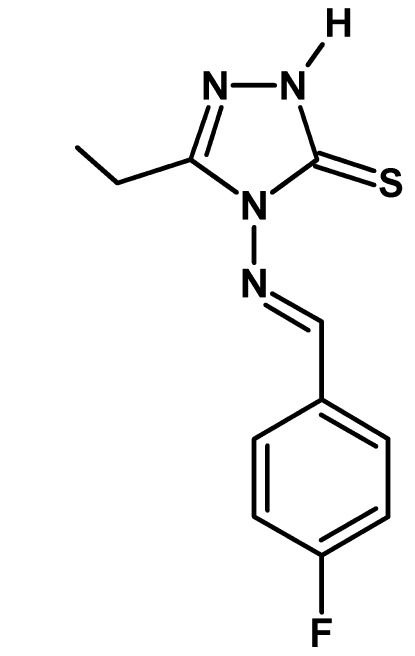


## Experimental

### 

#### Crystal data

C_11_H_11_FN_4_S
*M* *_r_* = 250.30
Monoclinic, 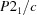

*a* = 7.7967 (17) Å
*b* = 8.4205 (19) Å
*c* = 19.138 (4) Å
β = 99.780 (4)°
*V* = 1238.2 (5) Å^3^
*Z* = 4
Mo *K*α radiation
μ = 0.26 mm^−1^
*T* = 293 K
0.20 × 0.20 × 0.15 mm

#### Data collection

Bruker SMART CCD diffractometer
Absorption correction: ψ scan (*SADABS*; Sheldrick, 2007 ▶) *T*_min_ = 0.770, *T*_max_ = 1.000
11403 measured reflections
2182 independent reflections
1586 reflections with *I* > 2σ(*I*)
*R*_int_ = 0.050

#### Refinement

*R*[*F* ^2^ > 2σ(*F* ^2^)] = 0.045
*wR*(*F* ^2^) = 0.115
*S* = 1.02
2182 reflections
154 parameters
H-atom parameters constrained
Δρ_max_ = 0.20 e Å^−3^
Δρ_min_ = −0.19 e Å^−3^

### 

Data collection: *SMART* (Bruker, 2001 ▶); cell refinement: *SAINT* (Bruker, 2001 ▶); data reduction: *SAINT*; program(s) used to solve structure: *SHELXS97* (Sheldrick, 2008 ▶); program(s) used to refine structure: *SHELXL97* (Sheldrick, 2008 ▶); molecular graphics: *ORTEP-3* (Farrugia, 1997 ▶); software used to prepare material for publication: *SHELXL97*.

## Supplementary Material

Crystal structure: contains datablock(s) I, global. DOI: 10.1107/S1600536812015346/hb6709sup1.cif

Structure factors: contains datablock(s) I. DOI: 10.1107/S1600536812015346/hb6709Isup2.hkl

Supplementary material file. DOI: 10.1107/S1600536812015346/hb6709Isup3.cml

Additional supplementary materials:  crystallographic information; 3D view; checkCIF report

## Figures and Tables

**Table 1:** Hydrogen-bond geometry (Å, °)

*D*—H⋯*A*	*D*—H	H⋯*A*	*D*⋯*A*	*D*—H⋯*A*
N4—H4⋯S1^i^	0.86	2.48	3.3275 (19)	168
C11—H11⋯S1	0.93	2.55	3.222 (3)	129

Symmetry code: (i) 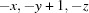.

**Fig. 1.**
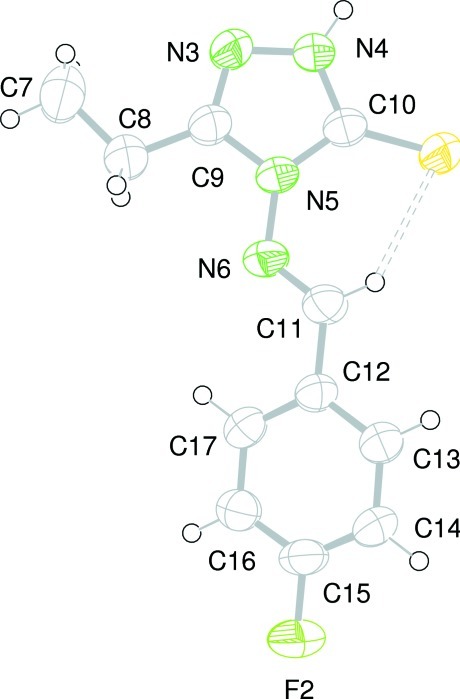
The molecular structure of the title compound. Displacement ellipsoids are drawn at the 50% probability level.

**Fig. 2.**
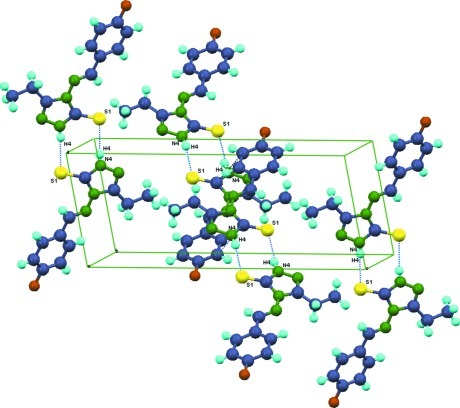
Paciking of the molecules.

## supplementary crystallographic information

### Comment

Earlier we reported the crystal structure of 1-{1-[2,8- Bis(trifluoromethyl)-4-quinolyl]-5-methyl-1*H*-1,2,3-triazol- 4-yl}ethanone (Devarajegowda *et al.*,2010). We report here the crystal structure of the title compound (Fig. 1). The packing of the molecules in the title structure is depicted in Fig. 2. The 1,2,4 triazole ring (N3 N4 N5 C9 C10 is not coplanar with the benzene ring (C12—C17) system; the dihedral angle between the two planes being 25.04 (12)°. The crystal structure is characterized by intermolecular N4—H4···S1 and intramolecular C11—H11···S1 interactions are observed (Table 1).

### Experimental

An equimolar mixture of the triazole (0.02 mol) and 4-fluorobenzaldehyde (0.02 mol) in absolute ethanol (30 ml) was refluxed with concentrated H_2_S_O_4 (0.5 ml) for 1–2 hrs. On cooling the reaction mixture, the solid product separated was crystallized from ethanol as colourless blocks. The synthesized compound was evaluated for antibacterial and antifungal activity by cup-plate diffusion method and used as the standard drugs for antibacterial and antifungal activity respectively.

### Refinement

All H atoms were placed at calculated positions and refined as riding, with N—H = 0.86 Å, C*sp*^2^—H = 0.93 Å, *C*(methylene)—H = 0.97 and *C*(methyl)—H = 0.96 Å. *U*_iso_(H) = *xU*_eq_(C,*N*), where *x* = 1.5 for methyl H and 1.2 for all other H atoms.

### Crystal data

**Table:**

C_11_H_11_FN_4_S	*F*(000) = 520
*M**_r_* = 250.30	*D*_x_ = 1.343 Mg m^−^^3^
Monoclinic, *P*2_1_/*c*	Melting point: 414 K
Hall symbol: -P 2ybc	Mo *K*α radiation, λ = 0.71073 Å
*a* = 7.7967 (17) Å	Cell parameters from 2182 reflections
*b* = 8.4205 (19) Å	θ = 2.2–25.0°
*c* = 19.138 (4) Å	µ = 0.26 mm^−^^1^
β = 99.780 (4)°	*T* = 293 K
*V* = 1238.2 (5) Å^3^	Block, colourless
*Z* = 4	0.20 × 0.20 × 0.15 mm

### Data collection

**Table:**

Bruker SMART CCD diffractometer	2182 independent reflections
Radiation source: fine-focus sealed tube	1586 reflections with *I* > 2σ(*I*)
Graphite monochromator	*R*_int_ = 0.050
ω and φ scans	θ_max_ = 25.0°, θ_min_ = 2.2°
Absorption correction: ψ scan (*SADABS*; Sheldrick, 2007)	*h* = −9→9
*T*_min_ = 0.770, *T*_max_ = 1.000	*k* = −10→10
11403 measured reflections	*l* = −22→22

### Refinement

**Table:**

Refinement on *F*^2^	Primary atom site location: structure-invariant direct methods
Least-squares matrix: full	Secondary atom site location: difference Fourier map
*R*[*F*^2^ > 2σ(*F*^2^)] = 0.045	Hydrogen site location: inferred from neighbouring sites
*wR*(*F*^2^) = 0.115	H-atom parameters constrained
*S* = 1.02	*w* = 1/[σ^2^(*F*_o_^2^) + (0.0551*P*)^2^ + 0.1712*P*] where *P* = (*F*_o_^2^ + 2*F*_c_^2^)/3
2182 reflections	(Δ/σ)_max_ < 0.001
154 parameters	Δρ_max_ = 0.20 e Å^−^^3^
0 restraints	Δρ_min_ = −0.19 e Å^−^^3^

### Special details

**Table:**

Geometry. All s.u.'s (except the s.u. in the dihedral angle between two l.s. planes) are estimated using the full covariance matrix. The cell s.u.'s are taken into account individually in the estimation of s.u.'s in distances, angles and torsion angles; correlations between s.u.'s in cell parameters are only used when they are defined by crystal symmetry. An approximate (isotropic) treatment of cell s.u.'s is used for estimating s.u.'s involving l.s. planes.
Refinement. Refinement of *F*^2^ against ALL reflections. The weighted *R*-factor *wR* and goodness of fit *S* are based on *F*^2^, conventional *R*-factors *R* are based on *F*, with *F* set to zero for negative *F*^2^. The threshold expression of *F*^2^ > 2σ(*F*^2^) is used only for calculating *R*-factors(gt) *etc*. and is not relevant to the choice of reflections for refinement. *R*-factors based on *F*^2^ are statistically about twice as large as those based on *F*, and *R*- factors based on ALL data will be even larger.

### Fractional atomic coordinates and isotropic or equivalent isotropic displacement parameters (Å^2^)

**Table:**

	*x*	*y*	*z*	*U*_iso_*/*U*_eq_	
S1	0.22343 (8)	0.61721 (9)	0.07429 (3)	0.0711 (3)	
F2	1.1525 (2)	1.1308 (2)	0.18960 (8)	0.0886 (5)	
N3	0.2231 (2)	0.5813 (2)	−0.12894 (9)	0.0579 (5)	
N4	0.1651 (2)	0.5520 (2)	−0.06628 (9)	0.0542 (5)	
H4	0.0729	0.4974	−0.0641	0.065*	
N5	0.3973 (2)	0.6845 (2)	−0.03736 (9)	0.0492 (5)	
N6	0.5370 (2)	0.7802 (2)	−0.00763 (10)	0.0536 (5)	
C7	0.4043 (4)	0.7150 (5)	−0.23453 (15)	0.1142 (13)	
H7A	0.4826	0.7624	−0.2622	0.171*	
H7B	0.2932	0.7669	−0.2449	0.171*	
H7C	0.3906	0.6043	−0.2460	0.171*	
C8	0.4763 (3)	0.7329 (4)	−0.15769 (12)	0.0706 (8)	
H8A	0.5896	0.6822	−0.1480	0.085*	
H8B	0.4929	0.8450	−0.1469	0.085*	
C9	0.3637 (3)	0.6635 (3)	−0.11018 (12)	0.0529 (6)	
C10	0.2640 (3)	0.6153 (3)	−0.00883 (11)	0.0512 (6)	
C11	0.5946 (3)	0.7675 (3)	0.05793 (13)	0.0555 (6)	
H11	0.5433	0.6957	0.0850	0.067*	
C12	0.7417 (3)	0.8648 (3)	0.09174 (11)	0.0499 (6)	
C13	0.8090 (3)	0.8408 (3)	0.16278 (12)	0.0648 (7)	
H13	0.7607	0.7634	0.1881	0.078*	
C14	0.9473 (3)	0.9307 (3)	0.19634 (12)	0.0689 (7)	
H14	0.9920	0.9155	0.2441	0.083*	
C15	1.0162 (3)	1.0415 (3)	0.15780 (13)	0.0594 (6)	
C16	0.9559 (3)	1.0674 (3)	0.08733 (12)	0.0569 (6)	
H16	1.0078	1.1429	0.0623	0.068*	
C17	0.8163 (3)	0.9789 (3)	0.05422 (12)	0.0521 (6)	
H17	0.7723	0.9960	0.0065	0.063*	

### Atomic displacement parameters (Å^2^)

**Table:**

	*U*^11^	*U*^22^	*U*^33^	*U*^12^	*U*^13^	*U*^23^
S1	0.0555 (4)	0.1101 (6)	0.0457 (4)	−0.0195 (4)	0.0028 (3)	−0.0027 (3)
F2	0.0719 (10)	0.1093 (13)	0.0787 (11)	−0.0335 (9)	−0.0044 (8)	−0.0223 (9)
N3	0.0501 (11)	0.0773 (14)	0.0440 (11)	−0.0060 (10)	0.0019 (9)	−0.0030 (10)
N4	0.0456 (10)	0.0688 (13)	0.0461 (11)	−0.0116 (9)	0.0020 (8)	0.0004 (9)
N5	0.0397 (10)	0.0599 (12)	0.0443 (11)	−0.0034 (9)	−0.0035 (8)	0.0005 (9)
N6	0.0432 (10)	0.0618 (12)	0.0517 (12)	−0.0058 (9)	−0.0035 (9)	−0.0017 (9)
C7	0.093 (2)	0.192 (4)	0.059 (2)	−0.030 (2)	0.0190 (17)	0.000 (2)
C8	0.0578 (15)	0.099 (2)	0.0547 (16)	−0.0130 (14)	0.0075 (12)	0.0037 (14)
C9	0.0456 (13)	0.0638 (15)	0.0466 (14)	0.0002 (11)	−0.0002 (10)	0.0019 (11)
C10	0.0416 (12)	0.0599 (14)	0.0488 (13)	−0.0004 (11)	−0.0023 (10)	0.0010 (11)
C11	0.0431 (12)	0.0637 (16)	0.0551 (15)	−0.0021 (11)	−0.0050 (11)	0.0101 (12)
C12	0.0409 (11)	0.0600 (15)	0.0462 (13)	−0.0001 (11)	−0.0002 (10)	0.0006 (11)
C13	0.0508 (14)	0.0891 (19)	0.0507 (15)	−0.0103 (13)	−0.0026 (11)	0.0105 (13)
C14	0.0545 (14)	0.103 (2)	0.0444 (14)	−0.0125 (14)	−0.0050 (11)	0.0015 (14)
C15	0.0436 (13)	0.0717 (17)	0.0591 (15)	−0.0072 (12)	−0.0017 (11)	−0.0137 (13)
C16	0.0531 (13)	0.0570 (15)	0.0601 (15)	−0.0048 (11)	0.0082 (11)	−0.0028 (12)
C17	0.0516 (13)	0.0573 (14)	0.0449 (12)	0.0041 (11)	0.0007 (10)	−0.0003 (11)

### Geometric parameters (Å, º)

**Table:**

S1—C10	1.674 (2)	C8—H8A	0.9700
F2—C15	1.358 (3)	C8—H8B	0.9700
N3—C9	1.295 (3)	C11—C12	1.467 (3)
N3—N4	1.374 (2)	C11—H11	0.9300
N4—C10	1.341 (3)	C12—C17	1.385 (3)
N4—H4	0.8600	C12—C13	1.387 (3)
N5—C10	1.382 (3)	C13—C14	1.383 (3)
N5—C9	1.385 (3)	C13—H13	0.9300
N5—N6	1.396 (2)	C14—C15	1.355 (3)
N6—C11	1.263 (3)	C14—H14	0.9300
C7—C8	1.490 (4)	C15—C16	1.368 (3)
C7—H7A	0.9600	C16—C17	1.381 (3)
C7—H7B	0.9600	C16—H16	0.9300
C7—H7C	0.9600	C17—H17	0.9300
C8—C9	1.487 (3)		
			
C9—N3—N4	104.05 (17)	N4—C10—S1	127.43 (17)
C10—N4—N3	114.55 (18)	N5—C10—S1	130.33 (17)
C10—N4—H4	122.7	N6—C11—C12	120.7 (2)
N3—N4—H4	122.7	N6—C11—H11	119.7
C10—N5—C9	108.50 (18)	C12—C11—H11	119.7
C10—N5—N6	132.02 (18)	C17—C12—C13	119.1 (2)
C9—N5—N6	118.99 (18)	C17—C12—C11	121.6 (2)
C11—N6—N5	118.52 (19)	C13—C12—C11	119.2 (2)
C8—C7—H7A	109.5	C14—C13—C12	120.7 (2)
C8—C7—H7B	109.5	C14—C13—H13	119.6
H7A—C7—H7B	109.5	C12—C13—H13	119.6
C8—C7—H7C	109.5	C15—C14—C13	118.3 (2)
H7A—C7—H7C	109.5	C15—C14—H14	120.9
H7B—C7—H7C	109.5	C13—C14—H14	120.9
C9—C8—C7	113.6 (2)	C14—C15—F2	119.3 (2)
C9—C8—H8A	108.8	C14—C15—C16	123.0 (2)
C7—C8—H8A	108.8	F2—C15—C16	117.7 (2)
C9—C8—H8B	108.8	C15—C16—C17	118.6 (2)
C7—C8—H8B	108.8	C15—C16—H16	120.7
H8A—C8—H8B	107.7	C17—C16—H16	120.7
N3—C9—N5	110.71 (19)	C16—C17—C12	120.3 (2)
N3—C9—C8	126.9 (2)	C16—C17—H17	119.9
N5—C9—C8	122.3 (2)	C12—C17—H17	119.9
N4—C10—N5	102.12 (18)		

### Hydrogen-bond geometry (Å, º)

**Table:**

*D*—H···*A*	*D*—H	H···*A*	*D*···*A*	*D*—H···*A*
N4—H4···S1^i^	0.86	2.48	3.3275 (19)	168
C11—H11···S1	0.93	2.55	3.222 (3)	129

Symmetry code: (i) −*x*, −*y*+1, −*z*.
